# *Anthrenus* sp. and an Uncommon Cluster of Dermatitis

**DOI:** 10.3201/eid2707.203245

**Published:** 2021-07

**Authors:** Loïc Simon, Fériel Boukari, Halilou Almou Oumarou, Thomas Hubiche, Pierre Marty, Christelle Pomares, Pascal Delaunay

**Affiliations:** Centre Hospitalier Universitaire de Nice, Nice, France (L. Simon, F. Boukari, H. Almou Oumarou, T. Hubiche, P. Marty, C. Pomares, P. Delaunay);; Université Côte d’Azur, Nice (L. Simon, P. Marty, C. Pomares);; Université de Montpellier, Montpellier, France (P. Delaunay)

**Keywords:** Anthrenus sp., carpet beetle, insects, insect pests, parasites, bed bugs, fleas, medical entomology, dermatitis, cluster, skin lesions, vector-borne infections, zoonoses, Nice, France

## Abstract

We report patients in their homes in France who had cutaneous lesions caused by *Anthrenus* sp. larvae during the end of winter and into spring. These lesions mimic bites but are allergic reactions to larvae hairs pegged in the skin. These lesions should be distinguished from bites of bed bugs or fleas.

Among all biting insects, some are responsible only for bite lesions, and others are also vectors of diseases (*1*–*3*). In both instances, these insects are a physical nuisance and sometimes a psychological one. The most-described biting insects found in human dwellings are bed bugs, which are transmitted by travel and movement of persons; and fleas, which are transmitted by household pets (*3*,*4*). We describe patients requesting a dermatologic consultation for skin lesions caused by hairs of *Anthrenus* sp. (carpet beetle) larvae.

## The Study

During January‒March 2020, a total of 11 patients (6 children 7‒17 years of age and 5 adults 19‒58 years of age) from 7 families living in southern France (Nice area) consulted with the Department of Dermatology, Centre Hospitalier Universitaire de Nice (Nice, France). Each person had a several-week history of multiple skin lesions. Clinical examinations showed 7‒35 (median 17) isolated erythematous urticarial papules/patient. These papules were pruritic, and lasted ≈1 week before disappearing slowly; new papules then appeared. Erythematous papular lesions always appeared first in 1 family member. Then, in all cases, dermatitis progressively affected some, but not all, family members. Lesions and absence of other symptoms did not evoke a specific dermatologic condition. Because the scattering of symptoms among family members was compatible with insect infestation, patients were referred to the Department of Medical Entomology for further examination.

**Figure 1:**
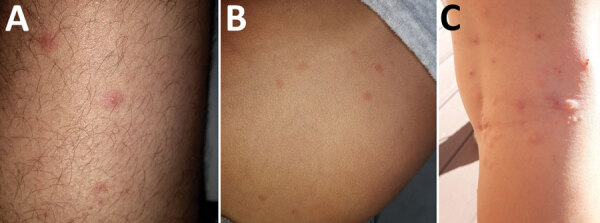
Aspects of lesions caused by larvae of *Anthrenus* sp. carpet beetles on 3 members of the same family, France. A) Thigh of a 33-year-old man; B) abdomen of a 5-year-old boy; C) leg of an 8-year-old girl (who scratched lesions).

**Figure 2:**
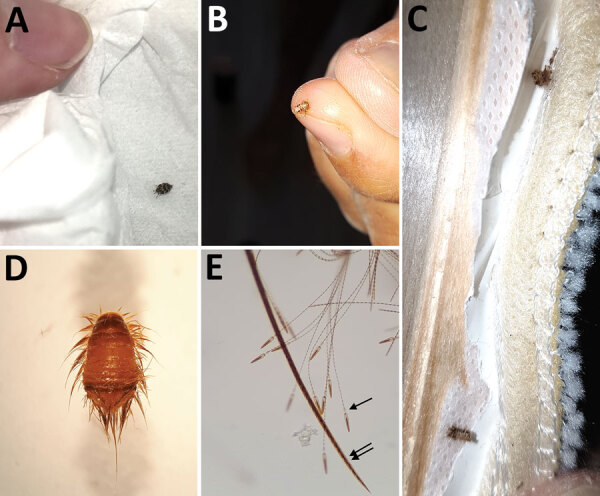
Stages of *Anthrenus* sp. carpet beetle. A) Adult stage (length 4 mm); B, C) larval stage (length 4 mm) found inside clothing and upholstery fabric; D) larvae (original magnification ×40); and E) larvae (original magnification ×200) showing fine hairs (single arrow) that have a spear-headed shape, are responsible for human hypersensitivity, and are invisible to the naked eye. Double arrow indicates thick larvae hair.

No recent history of travel, purchase of second-hand items, or presence of infected pets (confirmed by veterinarians) were reported by the families. Examinations indicated that lesions were located mostly under clothing: on the thighs, arms, chest, and abdomen (Figure 1). Careful inspection of beds and sofas by the families did not find bed bugs or fleas. In addition, the fact that the lesions were scattered all over the body, mainly under clothing, was not typical for bed bugs and fleas (Table) (*4*). In this context, the medical entomologist visited 2 homes and looked for mites or insects responsible for the dermatitis. He confirmed the absence of bed bugs and fleas, and rapidly found larvae and adult insects in clothing, fabric, and upholstery inside the homes (Figure 2, panels A‒C). These larvae and adult insects were later identified as specimens of *Anthrenus* sp. by using a 40× binocular magnifier (Figure 2, panel D). For the other families, he asked the patients to look for *Anthrenus* sp. in these same places, showing them pictures of carpet beetle adults and larvae (*5*). All families found similar larvae and adult insects and brought them to the medical entomologist, who confirmed *Anthrenus* sp. by morphology.

**Table:** Characteristics of 3 insects found in dwellings during a study of *Anthrenus* sp. and an uncommon cluster of dermatitis*

Insect pest	Configuration of skin lesions			Time of year	Harmful stage of insect	Location in housing	Treatment for housing	Evolution without treatment
Body part affected	Location in clothes	Grouping
Bed bugs	Face, hands, feet	Uncovered areas	Frequently 3 or 4	Any season	All	Beds, sofas	Steam >60°C with or without insecticide	Exponential
Fleas	Buttocks, legs	Covered or uncovered areas	Frequently 3 or 4	Any season	Adult	Adults: animals; larvae: carpets, sofas	Animal treatment, vacuum carpets and sofas	Depending on presence of infected animal
*Anthrenus* sp.	No specific parts	Mostly covered areas	Isolated	Late winter, early spring	Larval	Baseboards, wardrobes, mattresses, old carpets, drawers	Vacuum and cleaning of air conditioning systems	Possible spontaneous healing at end of spring

*Treatment for all patients was antihistamines and topical corticoids.

*Anthrenus* sp., better known as the carpet beetle, belongs to the order Coleoptera and family Dermestidae. Adults have a length of ≈3–4 mm, feed on nectar and pollen, and are harmless to humans. During autumn, female beetles search for hot areas and lay their eggs in dark places and cracks, making furniture one of their favorite spots (*6*,*7*). The larvae, which have a length of 4 mm, hatch at the end of winter or during spring. They usually live in dark drawers and cupboards. Larvae feed on dried organic matter from plant or animal residues, such as wool, dust, dead skin cells, feathers, and hairs; thus, they are commonly found in wardrobes, on stuffed animals, mattresses, or under carpets (*6*,*7*). Larvae never infest living animals. The larvae of many species of carpet beetles are covered with spear-headed hairs. These hairs are disseminated throughout the interior of a home by a natural air stream or an air conditioning system.

We observed for 1 child from the first family a large number of lesions (35 papules), probably caused by the air conditioning unit located above her bed. Two kinds of hairs coexist on these insect larvae. One hair is fine and has a terminal arrow, and the other hair is thick and has scales (Figure 2, panel E). Among the hairs of the *Anthrenus* sp. larvae, only the fine prickly hairs are responsible for lesions. Their spear-headed shape enable them to get stuck in the skin or respiratory mucosa, leading to hypersensitivity reactions in the hosts (*8*). Few cases of dermatitis caused by *Anthrenus* sp. beetles have been described (*6*,*9*,*10*). Even rare cases of asthma could be linked to the presence of carpet beetle larvae in the house (*11*).

Several steps (excluding insecticides) were recommended to quickly help the 7 families eliminate their lesions and clean their homes. All patients were given antihistamines and topical corticoids. Clothing that had direct skin contact and was to be worn in the next few days was washed to eliminate larvae hairs and stored after drying in airtight bags to protect them from the environment. In the homes, places where *Anthrenus* sp. beetles were found were inspected and cleaned. Insects were removed mechanically. Mattresses and other infested areas were vacuumed, and the vacuum bag was put in a plastic bag in the trash. Air conditioning systems present in the infested rooms were cleaned to prevent larvae hairs from spreading.

After observance of the above protocol, skin lesions healed in all affected adults and children in 3 days. A month later, no other lesions were observed in the 7 families.

## Conclusions

In our medical experience, infestations by *Anthrenus* sp. beetles have been sporadic. We observed a large number of cases during a short period, and a new case of *Anthrenus* sp. infestation was being investigated when this manuscript was being written. The families described in this report lived either in houses or apartments. They were not geographically near each other, but they all lived near parks or green spaces in urban or periurban areas.

Proper detection and identification of specimens is a key step in controlling insect pests. *Anthrenus* sp. larvae are responsible for allergic cutaneous reactions (not bites) caused by hairs hooked in the skin, leading to lesions found under clothing in members of the same household. The environment should be investigated for this carpet beetle in the case of skin lesions mimicking arthropod bites without a central blister. Reactions to larvae hairs are different from 1 family member to another, and >1 of these family members frequently have no symptoms.

Dermatitis caused by *Anthrenus* sp. larvae is underdiagnosed or confused with dermatitis caused by bed bugs or fleas. These erroneous diagnoses can lead to use of insecticides and thus to unnecessary, tiring, expensive, and toxic procedures. Dermatitis caused by *Anthrenus* sp. larvae has clinical and environmental characteristics relevant to ruling out other entomologic causes. It is useful to know that lesions caused by this insect are isolated and located mostly under clothing. Also, these insects are found in late winter or during spring, and bed bugs or fleas are not found contemporaneously. This insect pest has been uncommon in human medicine and theses cases could indicate its emergence. Physicians and dermatologists should be better aware of this insect.
